# Microvascular Skeletal-Muscle Crosstalk in Health and Disease

**DOI:** 10.3390/ijms241310425

**Published:** 2023-06-21

**Authors:** Gerald J. Pepe, Eugene D. Albrecht

**Affiliations:** 1 Department of Physiological Sciences, Eastern Virginia Medical School, Norfolk, VA 23501, USA; pepegj@evms.edu; 2 Department of Obstetrics, Gynecology and Reproductive Sciences, University of Maryland School of Medicine, Baltimore, MD 21201, USA; 3 Department of Physiology, University of Maryland School of Medicine, Baltimore, MD 21201, USA

**Keywords:** skeletal muscle, fetus, microvasculature, cross-talk, diabetes, hypertension, insulin resistance

## Abstract

As an organ system, skeletal muscle is essential for the generation of energy that underpins muscle contraction, plays a critical role in controlling energy balance and insulin-dependent glucose homeostasis, as well as vascular well-being, and regenerates following injury. To achieve homeostasis, there is requirement for “cross-talk” between the myogenic and vascular components and their regulatory factors that comprise skeletal muscle. Accordingly, this review will describe the following: [a] the embryonic cell-signaling events important in establishing vascular and myogenic cell-lineage, the cross-talk between endothelial cells (EC) and myogenic precursors underpinning the development of muscle, its vasculature and the satellite-stem-cell (SC) pool, and the EC–SC cross-talk that maintains SC quiescence and localizes ECs to SCs and angio-myogenesis postnatally; [b] the vascular–myocyte cross-talk and the actions of insulin on vasodilation and capillary surface area important for the uptake of glucose/insulin by myofibers and vascular homeostasis, the microvascular-myocyte dysfunction that characterizes the development of insulin resistance, diabetes and hypertension, and the actions of estrogen on muscle vasodilation and growth in adults; [c] the role of estrogen in utero on the development of fetal skeletal-muscle microvascularization and myofiber hypertrophy required for metabolic/vascular homeostasis after birth; [d] the EC–SC interactions that underpin myofiber vascular regeneration post-injury; and [e] the role of the skeletal-muscle vasculature in Duchenne muscular dystrophy.

## 1. Introduction

Skeletal muscle accounts for approximately 40% of body mass, and more than 25% of the cardiac output flows through skeletal muscle in the resting state ([1,2] for review). It is not surprising, therefore, that skeletal muscle is highly vascularized and contains the highest number/density of capillaries of any tissue ([3,4] for review). As an organ system, skeletal muscle is not only essential for locomotion and the generation of requisite metabolic energy in the form of ATP, which underpins muscle contraction/force generation, but also plays a critical role in controlling overall energy balance and insulin-dependent glucose homeostasis as well as vascular well-being, including blood pressure. In addition, skeletal muscle exhibits an incredible ability to regenerate following trauma [1].

**Figure 1:**
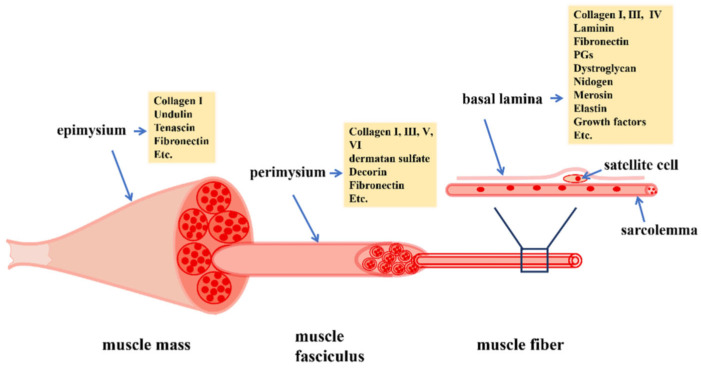
Overview of skeletal-muscle structure. Muscle fibers are comprised of multiple myofibrils surrounded by a layer of extracellular matrix (ECM), the basal lamina, and are grouped together to form a muscle fasciculus that is encapsulated by the perimysium. Several fascicles surrounded by the epimysium form the muscle mass. The ECM is comprised of collagens, proteoglycans and fibronectin. Myogenic stem/satellite cells reside below the basal lamina and play a vital role in muscle regeneration. From Korthuis, 2011 [1].

As shown in Figure 1 [1], skeletal muscle comprises several bundles of myofibers, each containing multiple myofibrils that contain mitochondria and contractile proteins, and surrounded by a layer of the extracellular matrix (ECM), the endomysium or basal lamina. Several bundles of myofibers are grouped together in a parallel arrangement to form a fascicle that is also encapsulated by a layer of the ECM, or perimysium, with several fascicles surrounded by another layer of the ECM, the epimysium, to form the muscle mass. ECM is a highly functional tissue comprised of collagens, proteoglycans and fibronectin, as well as proteins, such as dystrophin, which connect to the contractile muscle protein actin. Skeletal muscle also comprises several other cells including myogenic stem cells, or satellite cells (SC), that reside below the endomysium/basal lamina, and interstitial extra-laminal multi-potent stem cells. This niche of cells/ECM plays a vital role in muscle development, regeneration and overall function and, thus, is often dysregulated in various muscle diseases ([2,5,6] for review). Moreover, as discussed in more detail in this review, SCs are strikingly close to capillaries and the SC number correlates with the capillarization of myofibers [7]. Evidence also supports the hypothesis that the juxta-vascular SCs reciprocally interact with endothelial cells during differentiation to support angio-myogenesis [7].

**Figure 2:**
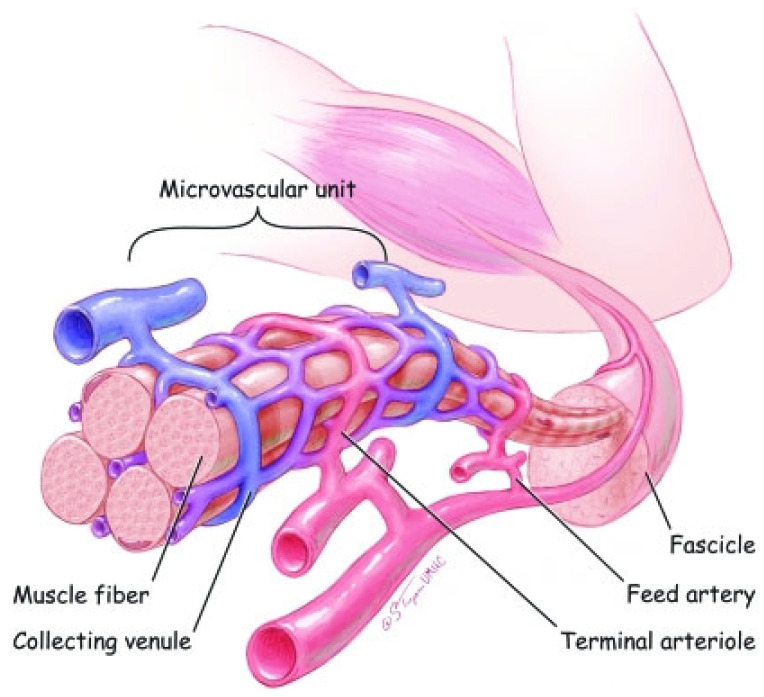
Diagram of skeletal-muscle vascular structure. Blood flow is provided by primary arteries, which give rise to feed arteries that course toward the epimysium. Smaller branching feed arteries, i.e., arterioles give rise to terminal arterioles that branch into numerous capillaries that travel parallel to the muscle fiber. Blood returns to collecting venules which merge to form larger venules. The terminal arterioles and all capillaries perfused by one terminal arteriole and collected by one venule define the skeletal-muscle microvascular unit (MVU), the smallest functional unit for blood-flow regulation in, and the site of uptake of, substrates, oxygen and regulatory factors by skeletal muscle fibers. From Korthuis, 2011 [1].

The vascular composition of skeletal muscle is extremely critical to overall muscle function as it is the conduit by which peripheral substrates (e.g., glucose) and regulatory factors (e.g., insulin) supporting muscle metabolism are made available. As outlined in Figure 2 [1], blood flow is provided by primary arteries that are distributed along the long axis of muscle and give rise to “feed” arteries that course toward the epimysium. Smaller branching feed vessels, i.e., arterioles, travel perpendicular to the muscle-fiber axis and give rise to terminal arterioles that penetrate the perimysium and then branch into numerous capillaries that are embedded in the endomysium and travel parallel to the muscle fiber. Blood returns to collecting venules which merge to form larger venules and are arranged in a manner similar to that in arterioles. The terminal arterioles, the last branches that contain vascular smooth muscle cells (VSMC), and all the capillaries perfused by one terminal arteriole and collected by one venule, define the skeletal-muscle microvascular unit (MVU). The MVU represents the smallest functional unit for blood-flow regulation in, and the site of uptake of, substrates, oxygen and regulatory factors by skeletal muscle. As a functional unit, the arterioles comprising VSMCs, endothelial cells and pericytes primarily regulate the perfusion and convective transport of substrates, the capillaries comprise endothelial cells and pericytes facilitate the majority of exchange through diffusion-mediated transport, and the venules act as collecting vessels [8]. As discussed by Latroche et al. ([9] for review) each MVU comprises 5–10 capillaries located in between 3–4 adjacent myofibers. Moreover, this overall vascular organization is similar in humans and small mammals [9,10], supporting the major role of capillary–muscle interactions in physiological processes [9].

Because of the multiple components (e.g., MVU, ECM, SC) that comprise the structure and support the function (e.g., metabolism, contractility) of skeletal myofibers, there is a requirement for precise coordination and “cross-talk” between these multiple components, as well as between the regulatory factors (e.g., hormones; growth factors) and metabolic substrates (e.g., carbohydrates) made available by other tissues and/or the environment. Elegant reviews on several aspects of skeletal-muscle structure and function and cellular cross-talk have been published [2,9,11,12]. This review will highlight the development and interaction of the MVU and skeletal myofibers, the impact of the intrauterine fetal hormonal milieu on the latter, and the importance of the MVU–skeletal-muscle interaction in metabolic diseases, including insulin resistance and type 2 diabetes and hypertension, and in muscle regeneration and the muscle disease Duchenne muscular dystrophy.

## 2. Development of Skeletal Muscle Myofibers and Microvasculature

Myogenic and vascular endothelial cells, as well as smooth muscle, are derived from a common embryonic structure, the dermomyotome (DMT), the dorsolateral part of the somite composed of mesodermal myogenic progenitor cells (MPC). An extensive body of the literature has examined, and ongoing studies are elucidating, the temporal sequence of the expression and mechanism(s) of action of the transcription factors that control the myogenic and vascular fate of the MPCs and, thus, the formation of muscle and its associated vasculature [5,13,14,15,16,17,18,19].

**Figure 3:**
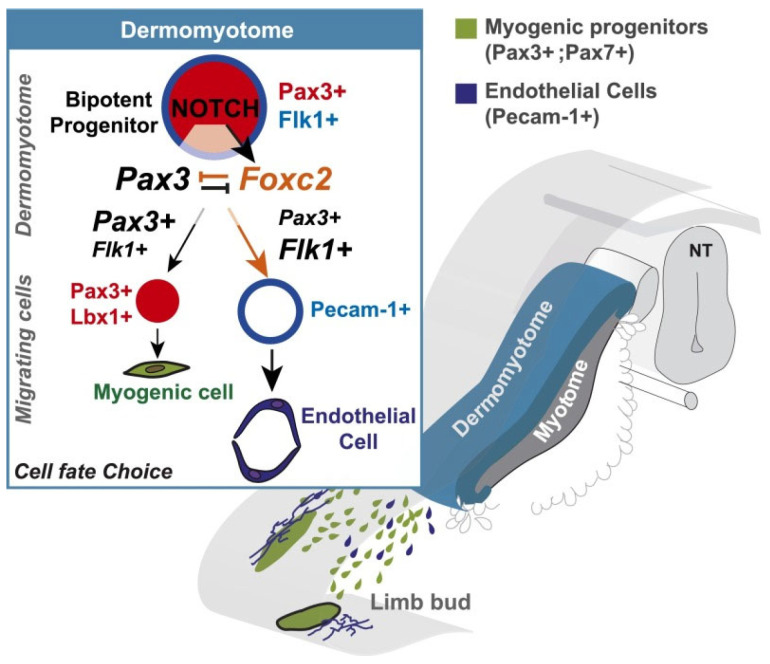
Schematic representation of the role of the Notch pathway in cell-fate decisions in the somite. Myogenic and vascular endothelial cells are derived from the dermomyotome comprising bipotent mesodermal myogenic progenitor cells (MPC) that express the transcription factors pax3^+^/pax7^+^ and foxc2. The myogenic–endothelial cell fate of MPC cells is regulated by the Notch signaling pathway via control of the pax3: foxc2 genetic equilibrium. From Mayeuf-Louchart et al., 2014 [17].

MPCs express the transcription factors pax3 and pax7, which function to control MPC myogenic specification. In the embryo, sequential waves of pax3^+^ and pax3^+^/7^+^ MPCs proliferate and migrate to the ventral surface of the DMT and form primary myofibers. The latter serve as scaffolding for formation of secondary myofibers, which occurs following the continued proliferation of MPCs and initiation of a highly orchestrated temporal expression of the myogenic regulatory factors including MyoD, Myf5, Mrf4 and the muscle differentiation factor MyoG (myogenin). However, MPCs also express the transcription factor foxc2, and Lagha et al. [15] showed that foxc2 expression was suppressed in pax3 gain-of-function mouse embryos and increased in pax3^−/−^ null mutants. Importantly, it is this apparent mutual repression between pax3^+^/7^+^ and foxc2 that determines the myogenic (high pax3^+^/7^+^:foxc2 ratio) or vascular (low pax3^+^/7^+^:foxc2 ratio) fate of MPCs. Studies in chick embryos also showed that Notch signaling promoted the formation of vascular smooth muscle at the expense of skeletal muscle [20]. Moreover, in the mouse embryo, Notch signaling is active in somites and the endothelial cells of blood vessels [21]. Accordingly, in an elegant study, Mayeuf-Louchart et al. [17] targeted one allele of the pax3 gene with a sequence coding the constitutively active intracellular domain of the Notch receptor to determine the role of Notch signaling in controlling the myogenic versus endothelial cell fate of MPC in mouse embryos. In Notch receptor gain-of-function embryos, the number of vascular smooth-muscle and endothelial cells derived from the somite (i.e., pax3^+^ MPC) was markedly increased, whereas MPC-dependent myogenesis was decreased. Moreover, fewer pax3^+^ cells were present in limbs, reflecting the promotion of an endothelial versus skeletal muscle cell fate of MPC. Additional somite transplant studies confirmed that the shift in MPC cell fate (i.e., the increased formation of endothelial cells) was associated with increased foxc2 expression whereas the inhibition of Notch signaling (i.e., increased myogenesis) decreased foxc2 expression. Therefore, as summarized in Figure 3, Mayeuf-Louchart et al. [17] concluded that the endothelial-myogenic cell fate of pax3^+^ MPC cells in the somite occurs before their migration to the limbs and is regulated by the Notch signaling pathway via control of the pax3: foxc2 genetic equilibrium.

Thus, as discussed by Latroche et al. [9], as a result of the coordinated Notch-signaling-dependent control of the pax3^+^/7^+^/foxc2 ratio in MPCs, a primary myotube surrounded by small blood vessels is formed very early in the embryo and serves as the scaffold for further muscle-vascular development. With advancing fetal development, an increased number of small blood vessels become localized throughout the greatly increased number of myofibers, a process termed secondary myogenesis and which is sustained by the continued proliferation of MPC myogenic cells as well as endothelial cells. Studies have also shown that the vascular invasion of myofibers participates in establishing the fascicular arrangement of the vascular bed in limb muscle. Interestingly, even in pax3^−/−^-null mice, animals that exhibit minimal if any myogenesis, angioblasts/endothelial cell precursors still invade the “myogenic space” and organize correctly. In addition to the pax3^+^/7^+^:foxc2 ratio, it is well established that vascular endothelial growth factor (VEGF) plays a requisite role in vasculogenesis in the embryo and the capillarization of tissue beds late in gestation [22,23]. VEGF is also an indispensable factor regulating the postnatal vascular maturation of tissue beds, including skeletal muscle, and accordingly capillary expansion was markedly decreased in VEGF-null skeletal muscle in mice [24,25,26]. Although myofiber number is established in mammals including humans in utero and is completed by birth, there is a marked increase in myofiber hypertrophy and a requisite increase in capillarization to support the increase in fiber size throughout early postnatal development ([27,28,29,30,31] for review). In addition, there is an architectural rearrangement of the microvascular bed into the characteristic pattern of long meshes that run parallel to the muscle fibers ([9] for review). Although the coordination and control of secondary myogenesis and concomitant vascular maturation including the formation of functional MVU in skeletal muscle in utero remain to be determined, studies by our group have recently documented an important role for estrogen, as discussed in Section 4 below.

**Figure 4:**
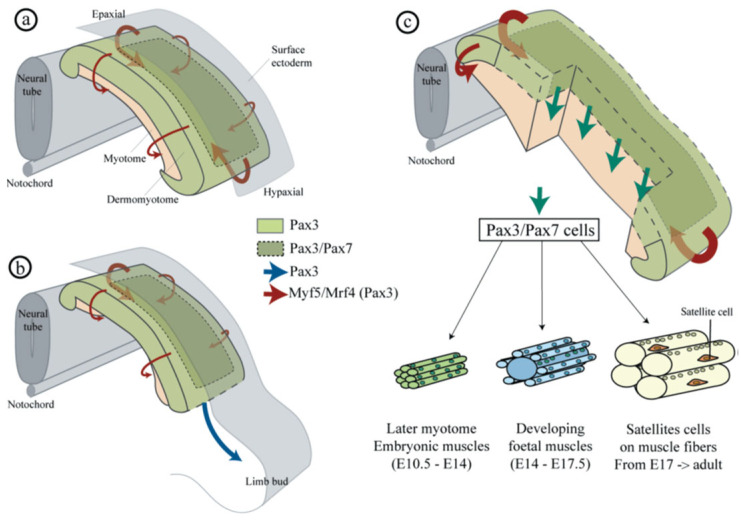
Model of myogenic proliferation and alignment of satellite cells (SC) under the basal lamina of skeletal muscle fibers. In the embryo, pax3^+^ and pax3^+^/7^+^ myogenic progenitor cells (MPC) proliferate and form primary myofibers which serve as scaffolding for the formation of muscle fibers, which occurs following continued proliferation of MPCs and expression of myogenic regulatory factors including MyoD, Myf5 and Mrf4. Proliferation of myogenic cells also leads to establishment of the pool of quiescent SC which do not proliferate nor enter the myogenic lineage. From Lagha et al., 2008 [14].

Finally, it is important to point out that the proliferation of myogenic cells is not only important to skeletal muscle development in the embryo/fetus, but also to the establishment of a myogenic-stem-cell pool comprised of pax3^+^ and/or pax7^+^ MPCs, and known as satellite cells (SC), which remain quiescent and do not proliferate nor enter the myogenic lineage. In the embryo, this population of myogenic stem cells becomes encapsulated by and resides under the basal lamina of developing myotubes (Figure 4) and in adults serves as the source of myogenic cells critical for regeneration of skeletal muscle myofibers following injury, as discussed in Section 5. Endothelial cells (EC) that invade the developing myotube in the fetus localize very close to the SC and thereby form an EC–SC niche, in which there is a bidirectional-reciprocal relationship between ECs and SCs. For example, in studies of the co-culture of ECs and SCs isolated from adult skeletal muscle, ECs secreted growth factors including VEGF, PDGF and FGF and promoted the proliferation of SCs and their differentiation into myoblasts [7]. Verma et al. [32] also showed that SCs express VEGF and that blocking VEGF action or SC VEGF formation via gene deletion significantly reduced EC–SC proximity, and proposed that SCs recruit capillary ECs via VEGF [32]. Finally, ECs via induction of Notch signaling and the activation of the downstream factor DII4 maintain SC quiescence [32], which is also promoted by angiopoietin-1 produced by vascular mural cells/pericytes [33,34]. Accordingly, there is considerable interaction and cross-talk between SCs and ECs in promoting myo-angiogenesis, cross-talk essential for the regeneration of skeletal muscle and associated vasculature post trauma [2,35,36,37,38] and most likely, but yet to be determined, in angio-myogenesis in the fetus (Table 1).

## 3. Vascular–Myocyte Interaction/Cross-Talk in Glucose and Vascular Homeostasis and Dysfunction in Diabetes and Hypertension

**Figure 5:**
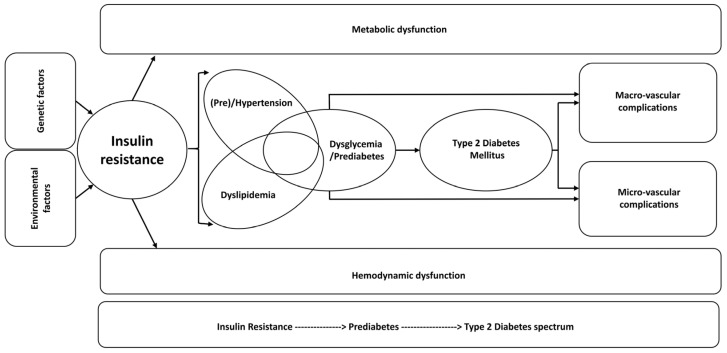
Key features of dysglycemia-based chronic disease and the insulin resistance–prediabetes–type 2 diabetes spectrum. Insulin resistance is the driving factor leading to prediabetes, diabetes, and micro- and macro-vascular complications. From Kalra et al., Diabetes, Metabolic Syndrome and Obesity: Targets and Therapy 2021:14 165–184. Originally published by and used with permission from Dove Medical Press Ltd. [43].

Type 2 diabetes mellitus (T2DM) has become a global epidemic and a significant public and economic health burden. It is estimated that 463 million people worldwide have T2DM and numbers are expected to increase to 578 million by 2030 and nearly 700 million by 2045 [41,42]. Moreover, a significant number of individuals exhibit dysglycemia or prediabetes, a condition that markedly increases the risk of developing T2DM and which is projected to affect 470 million people by 2030 [43,44]. In addition, individuals with T2DM and prediabetes have a greater risk of developing target-organ (e.g., cardiovascular, eye, kidney) damage. Although hyperglycemia is a major contributing factor, it appears that vascular dysfunction in general and hypertension in particular play important roles as well [42]. It is not surprising, therefore, that T2DM and hypertension often coexist and likely act synergistically to promote multiple clinical pathologies. Interestingly, studies indicate that the development of T2DM is 2.5 times more likely in euglycemic individuals with preexisting hypertension [42,45]. As pointed out in the 2015 Global Burden of Diseases, Injuries and Risk Factors study, high fasting glucose is the third and hypertension the number one most common global risk factor for disability-adjusted life years [42]. Finally, as summarized in Figure 5, evidence indicates that insulin resistance (IR) underpins, and is the precipitating factor in, the development of the disease spectrum of prediabetes, T2DM, and micro- and macro-vascular complications, now termed dysglycemia-based chronic disease (DBCD) by the American Association of Clinical Endocrinologists [46].

As outlined in Section 1, the microvasculature plays a fundamentally important role in regulating blood pressure and the delivery of substrates and regulatory factors to target cells, notably the myofibers that constitute skeletal muscle, which contain more capillaries than any other tissue [3,4]. Moreover, because of its mass and continued requirement for metabolic energy, skeletal muscle accounts for 80% of total insulin-directed glucose uptake and metabolism [47,48]. Importantly, insulin acts both on skeletal muscle myocytes and the microvasculature to assure metabolic as well as vascular homeostasis [9,49].

[a] Action of insulin on myocyte metabolism:

As outlined in the extensive review of Petersen and Shulman [50], insulin binds to its receptor on the myocyte membrane and activates a cascade of events that leads to the translocation of the glucose transporter protein (GLUT) 4 from the cytosol to the cell membrane, required for and resulting in the internalization of glucose from the intracellular fluid/interstitium into the cell. Internalized glucose is phosphorylated, and the coordinated action of insulin-receptor downstream signaling and glucose-6-phosphate on enzymes controlling glycogen turnover assures the availability of glucose for utilization, ultimately by mitochondria, as well as the storage of unused substrate as glycogen. Mitochondria possess their own genome which includes genes that comprise the respiratory-chain enzyme complexes and the coupling of oxidative phosphorylation/ATP production and electron transfer. Physiological bioenergetics is also dependent on mitochondrial biogenesis and mitophagy as well as the maintenance of a normal size, shape and distribution of mitochondria, which is coordinated by processes termed fission and fusion. Importantly, mitochondrial function is also influenced by the level of Ca^2+^ in the matrix and, when present in excess, leads to the opening of the mitochondrial permeability transition pore (MPT) [51]. The absolute requirement for the insulin receptor and the phosphorylation of insulin receptor substrate (IRS)-1 in the uptake and metabolism of substrate was demonstrated in insulin-receptor knockout and IRS1 knock-down in mice ([50] for review). Based on a significant body of the literature, it appears that myocyte IR reflects defects at the most proximal levels of insulin signaling, namely the insulin receptor and IRS1 and, thus, the malfunction of phosphatydilinsoitol-3 kinase (PI3K) and AKT serine-threonine kinase 2 (AKT2) [50]. As discussed by Petersen and Shulman [50] and Belosludtsev et al. [51], in addition to impaired uptake of glucose, mitochondrial function is also markedly altered in skeletal muscle in insulin resistance and T2DM. Dysfunction includes impaired mitochondrial biogenesis, the reduced activity of mitochondrial enzymes and enzyme complexes, decreased ATP synthesis, disturbed Ca^2+^ homeostasis, and the opening of MPT pores leading ultimately to cell death.

**Figure 6:**
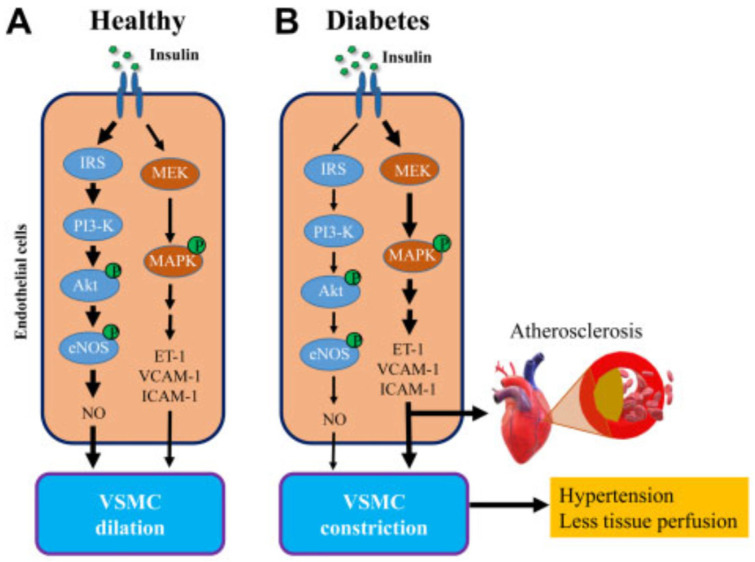
Endothelial insulin signaling and the associated consequences in health (**A**) and diabetes (**B**). In non-diabetic/healthy individuals, insulin binds to its membrane receptor to preferentially activate, via the insulin receptor substrate (IRS), the phosphatidyl inositol 3-kinase (PI3-K) pathway leading to the induction of endothelial nitric oxide synthase (eNOS), increased production of nitric oxide (NO) and vascular smooth muscle (VSMC) dilation. In diabetes, insulin-induced activation of PI3-kinase and NO production is compromised whereas induction of MAP-kinase is maintained, leading to production of endothlin-1 (ET-1), VSMC constriction and decreased myofiber perfusion and hypertension. From Love et al., 2021 [54].

[b] Action of insulin on MVU:

As reviewed by Zheng and Liu [52], blood vessels regulate blood pressure and tissue perfusion via the production and action of vasoactive factors in response to neural signals as well as vasoactive hormones. It is well established that insulin is a vasoactive hormone and acts on [a] large conduit arteries to increase vascular compliance, [b] the arterioles to increase vasodilation, reduce resistance and thus increase overall blood flow to tissue, and [c] on the precapillary arterioles to increase the capillary perfusion of the muscle MVU. Vascular endothelial cells express insulin receptors, and insulin enhances vasodilation by activating PI3K-regulated endothelial nitric oxide synthase (eNOS) activity, leading to the increased production of the vasodilator and vasoprotective factor nitric oxide (NO). Insulin action also results in stimulation of the mitogen-activated protein kinase pathway (MAPK), which controls vascular cell proliferation and the expression of adhesion molecules (e.g., VCAM) and the vasoconstrictor endothelin-1 (ET-1) [53,54]. The cross-talk elicited by insulin on these two signaling pathways (Figure 6) functions to modulate vascular tone, vascular cell proliferation and platelet aggregation to the endothelium [53,54].

**Figure 7:**
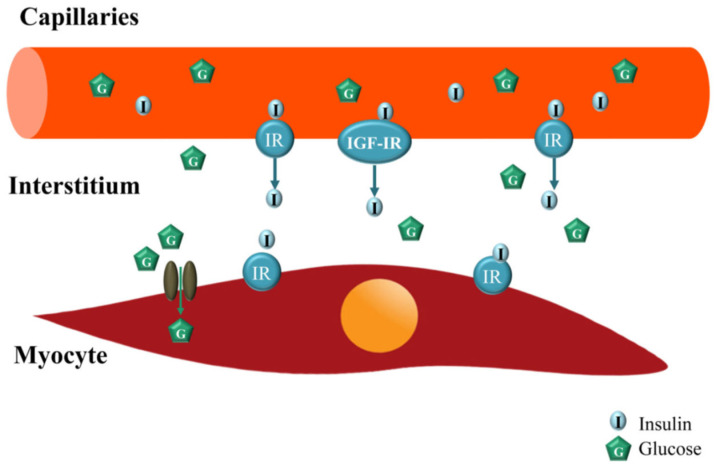
Diagram of trans-endothelial transport of insulin in skeletal muscle. Insulin in capillaries perfusing skeletal muscle binds to and activates endothelial cell insulin receptors (IR) and IGF-I receptors (IGF-IR) to activate PI3 kinase and MEK signaling pathways that facilitate trans-endothelial transport of insulin from blood to muscle interstitium and, thus, its availability to bind to and stimulate insulin myocyte receptors. From Zheng and Liu, 2015 [52].

**Figure 8:**
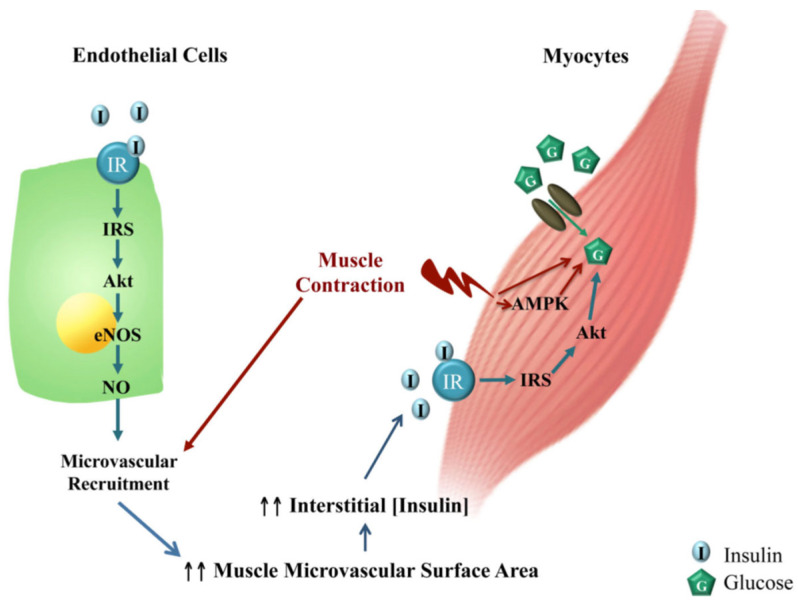
Schematic representation of the proposed interplay between exercise, insulin action and muscle microvasculature. Insulin stimulates endothelial-cell nitric oxide (NO) production which causes microvascular recruitment and expands endothelial surface area, leading to increased delivery to, and action of, insulin on muscle. Muscle contraction, via AMPK-dependent and -independent mechanisms, facilitates muscle-glucose and insulin uptake as well as NO-independent microvascular recruitment. From Zheng and Liu, 2015 [52].

As previously indicated, skeletal muscle is a major insulin-response organ important for overall glucose homeostasis. However, in order to act on its myocyte receptor, insulin must be delivered to the capillaries nourishing the myocyte, then transported through the capillary endothelium to enter the interstitial space/interstitium. Because it is the level of insulin in the interstitium, and not the plasma, that determines insulin’s metabolic action [54,55], studies have focused on determining the role of insulin on the skeletal muscle MVU. In a series of elegant studies, Barrett et al. [56,57,58] and others ([52,59,60] for review) established that the skeletal muscle MVU is an insulin target tissue and that insulin action at this site is closely coupled with its metabolic effects, i.e., myocyte glucose uptake. Accordingly, via its vasodilatory actions on the muscle MVU which increases capillary perfusion, insulin regulates its own trans-endothelial transport delivery to, and thus actions in, muscle myocytes (Figure 7) [52,61,62,63,64,65,66,67]. The regulation of the MVU by insulin is extremely important physiologically since even a relatively small increase in capillary perfusion in response to the insulin-induced vasodilation of precapillary terminal arterioles redirects blood flow from non-nutritive to nutritive myocytes, a process termed microvascular or capillary recruitment [56,68], and thereby increases the amount of substrate as well as insulin made available to the myocyte [52,69]. Importantly, insulin-regulated capillary recruitment occurs within 5–10 min and precedes insulin-stimulated glucose disposal [61,70,71]. Moreover, the blockade of insulin’s microvascular action by the eNOS inhibitor L-NAAME decreased insulin-stimulated steady-state glucose disposal by more than 40% [71,72,73]. This physiological interplay between insulin action on the MVU and myocytes is embellished in exercising muscle. As shown in Figure 8, in addition to an insulin–NO-dependent increase in capillary recruitment, there is an apparent NO-independent increase in recruitment of the MVU and thus enhanced capillary surface exchange following light–moderate exercise that does not increase blood flow to the conduit arteries, an effect that also results in the increased uptake of glucose and insulin and increased insulin action in myocytes ([52,74] for review). The latter in part underpins the value and role of exercise in controlling glucose homeostasis in prediabetics and early onset Type 2 diabetics.

[c] MVU myocyte dysfunction in type 2 diabetes:

It is not surprising that endothelial insulin resistance and dysfunction are present in skeletal muscle in individuals with T2DM [54]. Moreover, based on the Maastricht Study, it appears that there is overall microvascular dysfunction in individuals with T2DM as well as prediabetes [75]. Thus, patients with T2DM exhibit impaired endothelium-dependent flow-mediated dilation (FMD) [76], as well as insulin-mediated NO-dependent vasodilation and hypertension [77,78,79]. Interestingly, although endothelial insulin resistance is characterized by a profound suppression of insulin action on the PI3K eNOS-NO pathway, the function of the MAPK pathway and thus ET-1 production appear to be preserved (Figure 6). Moreover, in T2DM as well as in obesity and obesity-induced insulin resistance, there is a marked reduction in skeletal-muscle capillary density, a process termed rarefaction [54,80,81,82,83,84,85,86]. Importantly, the extent of capillary rarefaction correlates with the severity of insulin resistance and likely results from the reduced expression of endothelial growth factor production, notably VEGF [54,87]. The combination of microvascular dysfunction and capillary rarefaction further decreases myocyte perfusion and the endothelial surface area available for the uptake of insulin as well as glucose. Because the microvasculature is a major determinant of vascular resistance, and rarefaction of the microvasculature is a major factor in the etiology of hypertension [88,89], it is not surprising that hypertension is a co-morbidity in type 2 diabetics ([8,90] for review).

Interestingly, a few studies have shown that skeletal-muscle capillarization is increased in middle-aged men with impaired glucose tolerance preceding the development of T2DM, and in mice with early stage obesity and insulin resistance [91,92,93]. Although capillarization is typically assessed using two-dimensional (2D) analysis of tissue cross-sections, which exhibits some limitations that apparently can be avoided using 3D approaches [92,94], these findings do not appear to reflect methodologic differences since 2D analyses were employed in two of the studies [91,93]. Thus, as suggested by Ugwoke et al. [95] the precise role of capillary formation/rarefaction in the onset of insulin resistance remains an important topic for investigation.

There are also gender differences in vascular dysfunction in type 2 diabetics and men are more affected by microvascular complications than women, although T2DM contributes significantly to morbidity and mortality in both sexes [54,96,97,98]. The apparent sex/gender differences likely reflect the role of estrogen on vascular function. Thus, the major ovarian estrogen, estradiol (E_2_), via its stimulatory effects on VEGF, has a well-established role in the formation and expansion of the capillary network as well as endothelial eNOS expression and NO formation in the uterus, breast and other adult tissues [99,100,101,102,103,104,105]. Estrogen receptor (ER)α is abundantly expressed in skeletal muscle [106] and ERα-null mice exhibit insulin resistance within skeletal muscle as well as the liver [107,108]. In humans and mice, mutation of the aromatase gene controlling estrogen formation results in insulin resistance and glucose intolerance [109,110,111,112]. Importantly, E_2_ stimulates insulin sensitivity and glucose tolerance in the skeletal muscle of adult mice [113,114], reverses the increased incidence of IR in postmenopausal women [115,116,117], and also restores insulin sensitivity and glucose metabolism in ovariectomized monkeys fed a high-fat diet [118].

In addition, estrogen also has apparent direct effects on myocytes and increases muscle mass in adult rodents [119,120,121], and maintains muscle mass and strength in aging females [119,122]. These actions of estrogen appear to be mediated by the activation of (ER)β [123], which is also expressed in skeletal muscle [106,124]. In addition, both the administration of the ERα agonist propylpyrazoletriyl and activation of AKT increase myocyte GLUT4 expression and glucose uptake in rodent skeletal muscle [125,126,127].

It is apparent that skeletal muscle function and its role in overall glucose homeostasis and the regulation of blood pressure reflect the interactive actions of insulin and vascular endothelial and muscle growth factors, notably VEGF. This interaction and cross-talk underpin myocyte capillarization, the activation of critical intracellular signaling mechanisms to elicit vasodilation important to blood flow dynamics and blood pressure, and the recruitment of capillaries to enhance surface area, required for the optimal transport of glucose and insulin across the endothelium and into the interstitium, and thereby making them available for uptake by and action on the myocyte. Dysfunctional interaction resulting from IR and the loss of appropriate signaling precipitate alterations in the MVU myocyte function, leading to the progression of vascular dysfunction/high blood pressure and/or prediabetes, diabetes and ultimately multiple end-organ damage.

## 4. Estrogen Regulation of Fetal Skeletal Muscle Myogenesis and Capillarization In Utero: Impact on Glucose Homeostasis, Insulin Sensitivity and Vascular Function in Adulthood

**Figure 9:**
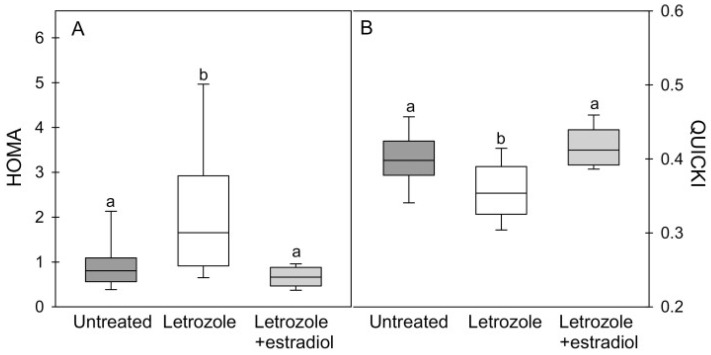
Box-and-whisker plots of values for (**A**) the homeostasis model of insulin resistance and (**B**) the QUICKI model of insulin sensitivity in offspring of baboons untreated (eight males, four females) or treated with letrozole (five males, three females) or letrozole plus estradiol (five males, three females). Values of data bars with different letters different from each other at *p* < 0.01 (ANOVA and Tukey–Kramer multiple comparison test). From Albrecht et al., 2022 [40].

As discussed above, E_2_ exhibits profound effects on skeletal-muscle vascular and myocyte function in adult animals and decreases the incidence of diabetes in postmenopausal women. In contrast to the numerous studies of estrogen action in adults, very little has been done to examine the role of the hormonal milieu in utero in general, and estrogen in particular, on the mechanisms integral to fetal development that prepare the offspring/adult for controlling insulin secretion and action after birth. Such studies are heightened by the extensive literature on the intrauterine programming of the fetal origin of adult diseases ([128,129,130] for review), and the fact that pregnancy in humans and non-human primates is characterized by a marked and sustained increase in maternal and fetal serum E_2_ levels during the second half of pregnancy [39]. Accordingly, the authors initiated studies using the nonhuman primate baboon model that our group developed to study the role of estrogen in pregnancy on insulin sensitivity/glucose homeostasis in adulthood [39]. To examine the latter, IV glucose tolerance tests were conducted in baboon offspring born to mothers untreated or treated daily with the aromatase inhibitor letrozole during the second half of gestation, which suppressed maternal and fetal E_2_ by >95%. As seen in Figure 9, male and female baboon offspring deprived of estrogen in utero exhibited abnormal glucose tolerance and IR, which was manifested prior to and persisted after the onset of puberty and into adulthood. Moreover, IR in post-pubertal animals was accompanied by onset of a deficit in first-phase insulin release, which other studies have shown leads to T2DM. Importantly, glucose intolerance and IR were prevented in offspring of animals treated in utero with letrozole plus E_2_ [40,131,132]. Studies also confirmed that IR in offspring deprived of E_2_ in utero was not due to an impairment of fetal or offspring growth, nor to any alteration in adipose or hepatic sensitivity to insulin [133]. Importantly, maternal glucose tolerance was normal in letrozole-treated mothers [40]. Moreover, the levels of skeletal-muscle insulin signaling molecules and glucose transporter proteins were unaltered in fetuses as well as in the offspring of estrogen-deprived baboons before and after glucose challenge [131,132,133]. Therefore, our group proposed that estrogen plays an important role in programming mechanisms in fetal skeletal muscle essential for glucose homeostasis and insulin sensitivity after birth [40,131,132].

**Figure 10:**
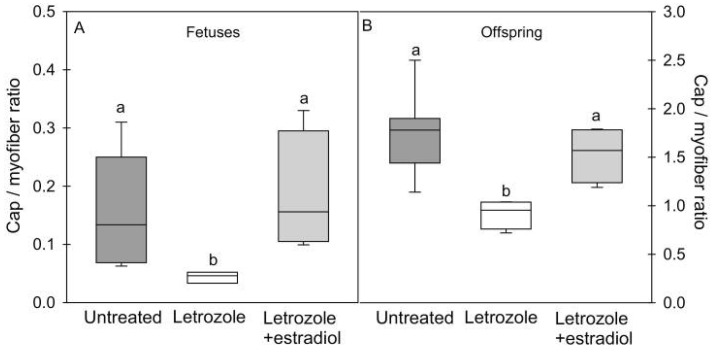
Box-and-whisker plots of (**A**) capillary (cap)/myofiber ratio (number of cap/number of myofibers) in fetuses (**A**) and offspring (**B**) delivered to baboon mothers untreated (*n* = 6 fetuses; *n* = 7 offspring) or treated with letrozole (*n* = 4 fetuses; *n* = 4 offspring) or letrozole plus estradiol (*n* = 4 fetuses; *n* = 4 offspring). Note the difference in *y*-axis scale between panels (**A**) (fetus) and (**B**) (offspring). Values with different letter superscripts differ from each other at *p* < 0.05, fetuses; *p* < 0.01, offspring (ANOVA Kruskal–Wallis multiple comparison test). From Albrecht et al., 2022 [40].

**Figure 11:**
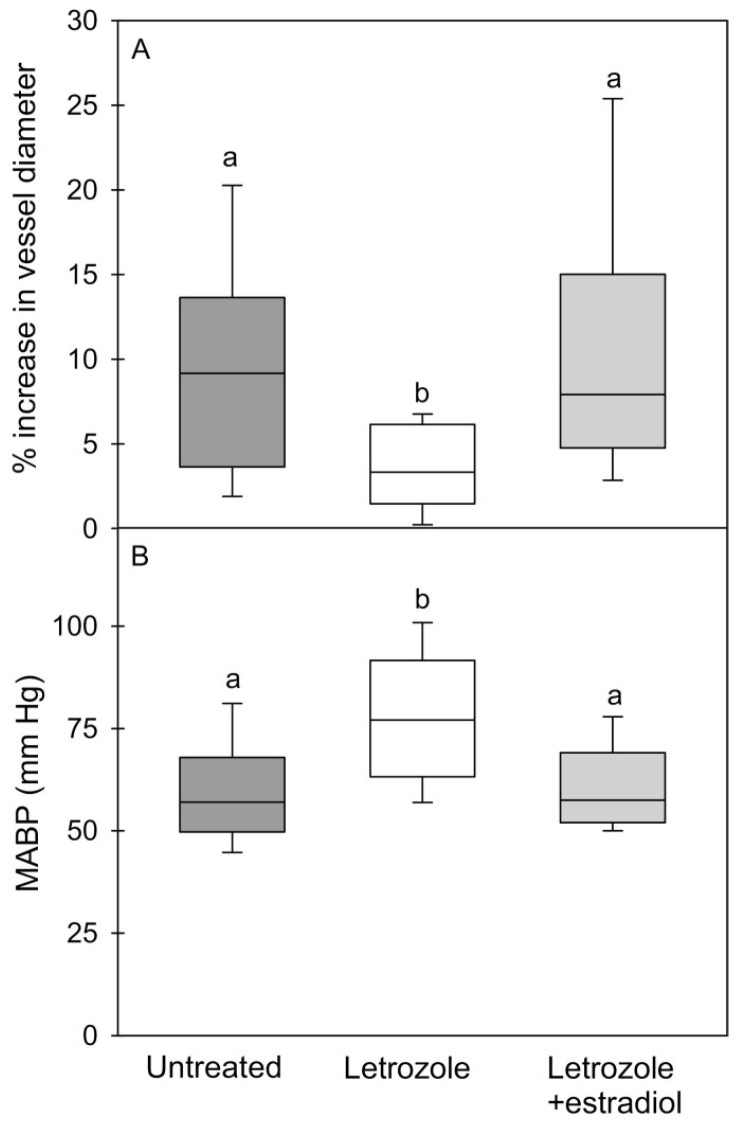
Box-and-whisker plots of the percentage increase (vs. basal) in brachial-artery diameter after shear stress (**A**) and mean arterial blood pressure (MABP) (**B**) in offspring delivered to baboons untreated (5 males, 7 females) or treated with letrozole (3 males, 6 females) or letrozole plus estradiol (3 males, 7 females). Values with different letter superscripts are different from each other (*p* < 0.01–*p* < 0.02, ANOVA and Kruskal–Wallis multiple comparison test). From Albrecht et al., 2022 [40].

Because of the important role of the MVU on myocyte function and the role of E_2_, directly and/or via the control of VEGF, on adult muscle and vascular function, our baboon model was used to examine whether estrogen regulated skeletal-muscle capillarization [40]. As seen in Figure 10, skeletal-muscle systemic micro-vascularization was markedly decreased in the fetus in E_2_-suppressed animals and restored to normal by treatment with E_2_. Moreover, the onset of impaired micro-vascularization elicited in the fetus by estrogen depletion was sustained in the offspring and accompanied by the onset of vascular endothelial dysfunction, manifested as a reduction in flow-mediated dilation and a marked increase in mean arterial blood pressure (Figure 11). Parallel studies also showed that in the absence of any change in fetal body weight, the size but not the number of fast and slow fibers in skeletal muscle was also markedly reduced in E_2_-suppressed baboons and restored to normal by concomitant treatment with letrozole plus estrogen [134]. The failure of myocytes to develop normally in E_2_-suppressed animals likely reflects the impairment of MVU/capillary development and/or an effect of estrogen on MPC-dependent hypertrophy of developing myofibers. Whether other aspects of muscle development were impaired, e.g., satellite cell pool, EC–SC interaction, remains to be ascertained.

**Figure 12:**
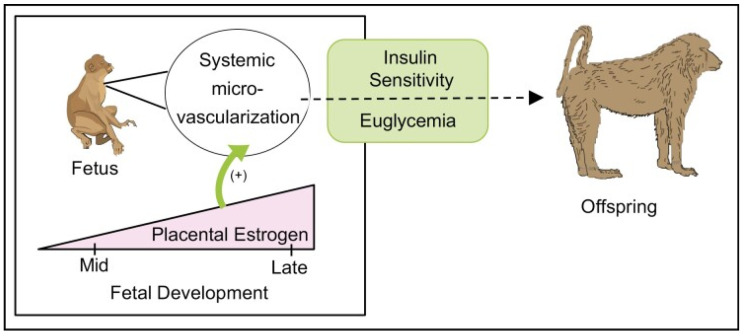
Proposed role of placental estrogen in promoting systemic microvascularization during fetal development and, thus, insulin sensitivity and euglycemia after birth in the offspring. From Albrecht et al., 2022 [40].

Collectively, these findings show that a well-developed systemic microvascular network in the primate fetus is essential for preparing target tissues, notably skeletal muscle, to respond to insulin after birth, and that a defect in microvessel development originating in the fetus precedes the onset of IR after birth [40]. Thus, the microvasculature is a potential target for early postnatal therapeutic strategies to prevent this developmental origin of disease. These findings are translatable to humans since children born premature, the incidence of which approximates 8–10% of all human pregnancies in the United States and curtails the exposure of the fetus to E_2_, exhibit microvessel rarefaction and IR [135,136,137,138]. Moreover, other clinical conditions that curtail the exposure of the fetus to E_2_, including preeclampsia, placental steroid deficiency and exposure to endocrine disruptors, are also associated with an increased risk of the development of IR and T2DM in offspring [139,140,141]. Studies of other researchers in animal models and humans have also shown that low birth weight elicited by various experimental paradigms, e.g., maternal nutrient restriction, hypoxia, and uterine-artery ligation, elicit IR in offspring ([31] for review), and likely reflects in large part the underdevelopment of skeletal muscle mass. However, as outlined above, in the E_2_-deprived baboon model, placental, fetal and offspring growth, maternal insulin sensitivity, and uterine and umbilical blood flow, as well as insulin-receptor signaling and liver and adipose function in offspring, were not altered by E_2_ deprivation, thereby establishing the important role of fetal exposure to estrogen. Thus, as summarized in Figure 12, the elevation in estrogen levels during the second half of primate pregnancy promotes microvascularization in the fetus and, thus, the normal development and function of the systemic vascular network and myofiber growth required for eliciting insulin sensitivity and glucose tolerance and normal blood pressure after birth [40].

## 5. Vascular–Myocyte and EC–SC Interaction/Cross-Talk in Muscle Repair/Regeneration

Skeletal muscle exhibits a remarkable ability to regenerate following trauma, a process dependent upon the activation of muscle SCs to rebuild/replace damaged and/or necrotic myofibers. As discussed in comprehensive reviews [33,142,143,144], muscle injury not only disrupts the multiple cell niches, e.g., EC–SC, that underpin muscle structure and function, but also leads to the infiltration of neutrophils and macrophages that release a battery of highly active inflammatory cytokines. Accordingly, the restoration of muscle myofibers involves a number of highly organized and interactive multi-cell processes, that occur over three phases termed [a] degeneration/inflammation: the rupture and necrosis of myofibers and an inflammatory response; [b] regeneration: the phagocytosis of damaged tissue and the activation of SCs leading to myofiber formation; and [c] remodeling: the maturation of new myofibers [142]. Concomitant with rebuilding myofibers, there is requirement for the restoration of damaged vasculature and MVUs to support myofiber growth as well as restore physiologic function, e.g., contractility and metabolism.

**Figure 13:**
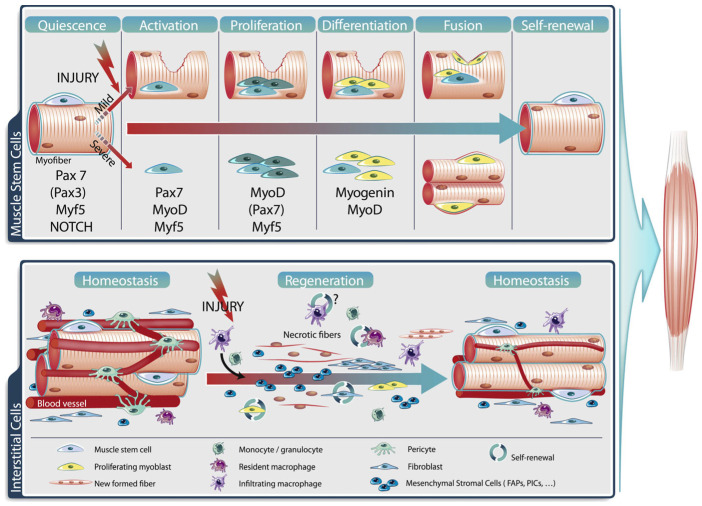
Synoptic view of muscle repair following mild injury, which induces fiber break and recruitment of satellite cells (SC) on surrounding intact myofibers, and severe injury, which triggers fiber destruction and SC proliferation on the extracellular matrix. Both mild and severe injury activate a tightly coordinated myogenic process and the interplay of key transcription factors. During homeostasis, SCs are quiescent and express Pax7 (and/or Pax3) and Myf5, and Notch signaling is active. Upon injury, SCs activate expression of the myocyte transcription factors MyoD and Myf5, and rapidly proliferate, and newly formed myoblasts express the terminal differentiation gene myogenin and exit the cell cycle. Differentiated myoblasts fuse to the pre-existing fibers (mild) or together to form new fibers (severe) while a population of SCs self-renew to replenish the stem-cell pool. Although the generation of new fibers is dependent on SCs, other cells, e.g., macrophages, monocytes, mesenchymal stromal cells, pericytes and fibroblasts also play a role in the regeneration process. From Baghdadi and Tajbakhsh, 2018 [143].

Muscle injury initiates lesions in the sarcolemma of the myofibrils, which leads to a marked increase in the levels of calcium and the activation of proteases and hydrolases that damage the myofibers and initiate an inflammatory response and the infiltration of neutrophils and macrophages. Injury also disrupts the EC–SC niche, leading to the activation of ECs and pericytes and the increased secretion of critical growth factors, including IGF-1 and VEGF, which along with increased levels of inflammatory cytokines of immune-cell origin, promote the symmetric division of committed myogenic SCs (e.g., pax7^+^/myf5^+^). The latter then undergo activation of the myofiber differentiation program and the expression of muscle regulatory factors, as essentially occurs during fetal development, and fuse to form new myofibers (Figure 13) [143]. A portion of the SCs are withdrawn from the cell cycle and restored to quiescence in the sub-laminar niche of new fibers, presumably by EC–SC cross-talk, and notably the activation of VEGF and DII4 by ECs. Depending on the extent of injury, myofiber regeneration usually peaks at about 2–3 weeks and diminishes thereafter [142]. Studies have also shown an important supportive role of other skeletal-muscle components, including fibro-adipogenic progenitors (FAP), which provide, via the secretion of key regulatory factors including Il-6, a beneficial microenvironment essential for optimal muscle regeneration ([33,145] for review). Interestingly, FAPs which reside in the interstitial space between myofibers can be problematic when dysregulated. For example, FAPs are believed to be key mediators of fatty- and fibrotic-tissue accumulation in the muscle of aging individuals, as well as in several muscular dystrophies, including Duchenne Muscular Dystrophy (DMD) and Amyotropic Lateral Sclerosis (ALS) ([33] for review).

It is well established that the release of VEGF by ECs, as well as by the SCs following muscle injury, promotes angiogenesis and capillary formation important for muscle regeneration ([144] for review). Accordingly, endothelial sprouts occur within 2–3 days post-injury and with the continued regeneration of capillary networks there is concomitant restoration of local perfusion by approximately day 5 [146,147,148]. At this juncture, however, the arteriolar networks are dilated, as the control of vasomotor tone and vasodilation and vasoconstriction is not established until approximately day 21 post-injury [146]. Although sprouting angiogenesis appears to be the mechanism by which vascular regeneration occurs following injury, vessel growth can also take place by intussusceptive or splitting angiogenesis [149,150]. Moreover, Egginton et al. [151] showed that capillary growth in rat skeletal muscle occurred by intussusceptive angiogenesis in response to increased blood flow and by sprouting angiogenesis in stretched muscle, and suggested that the type of angiogenesis was determined in large part by the site of the stimulus, i.e., intraluminal or abluminal. Thus, although both processes may function in vivo, it remains to be determined whether intussusceptive angiogenesis is also operative in regenerating skeletal muscle.

**Figure 14:**
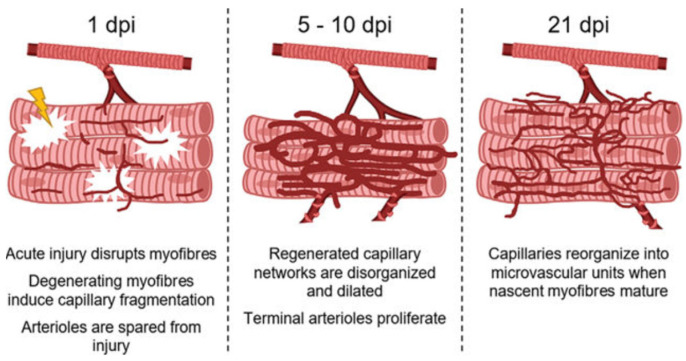
Model of microvascular injury and regeneration and remodeling of microvascular units after acute trauma to skeletal muscle. From Jacobsen et al., 2022 [148].

The revascularization of injured muscle and myofiber regeneration have been fairly well studied [9,152]. In contrast, less is known about how the microcirculation per se is affected by myofiber injury and recovery. Accordingly, in a recent study, Jacobson et al. [148] examined the time course of microvascular remodeling during myofiber regeneration following injury induced in mice by the myotoxin BaCl_2_. As summarized in Figure 14, within 24h of injury, capillary fragmentation was extensive, occurred in the presence or absence of neutrophils and coincided with myofiber degeneration. Accordingly, the authors proposed that myofiber degeneration most likely induced capillary fragmentation [148]. Interestingly, in BaCl_2_-damaged muscle, the arteriolar and venule networks remained intact. Moreover, in vitro studies showed that although BaCl_2_ induced a marked increase in calcium in microvessels isolated from uninjured muscle, SMCs and ECs did not exhibit cell death, unlike that induced by high calcium levels in myofibers. By day 5 post-injury, perfused capillary networks had reformed in association with the proliferation of terminal arterioles which continued through day 10. Interestingly, at day 10 and in the ensuing days post-injury there was minimal change in microvascular area in the capillary networks of regenerating myofibers. Moreover, only a few capillaries were aligned with the myofibers and thus were not organized into the MVU. Thus, capillary orientation and MVU organization did not occur until 21 days post-injury. Accordingly, the authors concluded that following their disruption secondary to myofiber damage, capillaries regenerate as disorganized networks that then undergo remodeling into functional MVUs as regenerated myofibers mature [148].

In summary, it is apparent that the regeneration of myofibers and the vascularity that underpins myofiber function involve the action/interaction of multiple cells, notably ECs, SCs and infiltrating immune cells, i.e., neutrophils and macrophages, as well as FAPs. ECs stimulate SC growth and SCs exhibit angiogenic properties, processes controlled by growth factors, i.e., VEGF and IGF-1. ECs and SCs, which are in close association with each other in an EC–SC niche, concomitantly proliferate after injury and cooperate to initiate SC myogenic commitment and myofiber regeneration, restore the SC stem-cell pool, reform the EC–SC niche, and enhance capillarization as well as the formation and remodeling of the functional MVU in regenerating muscle.

## 6. Vascular- Myocyte Dysfunction in Duchenne Muscular Dystrophy (DMD)

DMD is characterized by the progressive wasting of muscles caused by a mutation in the gene encoding the myofiber protein, dystrophin [9,153]. As a component of the dystrophin–glycoprotein complex (DGC), dystrophin connects the basal lamina of the ECM to the inner actin-based cytoskeleton and thereby reinforces the sarcolemma and prevents contraction-mediated muscle degradation. Accordingly, dystrophin deficiency leads to progressive myofiber damage and the replacement of muscle tissue by fibrotic or fibro-adipose tissue ([33,153] for review). Moreover, the myopathy of DMD is accompanied by a marked alteration in the morphology and function of mitochondria, including decreased oxidative phosphorylation, the enhanced generation of reactive oxygen species, and an opening of the MTP pore, effects leading to mitoptosis and myocyte apoptosis [154,155]. Interestingly, most of the injured muscle fibers in DMD occur in small clusters similar to that elicited in experimentally induced muscle infarcts. Thus, the myofiber necrosis characteristic of the disease was originally attributed to ischemia, i.e., a vascular problem [156], an hypothesis not fully pursued once the dystrophin gene/protein was isolated. However, dystrophin is also expressed in VSMC and, thus, a deficiency of the protein at this site could result in angiopathy. Indeed, in a dog model of DMD, animals exhibited a decrease in muscle capillary density and an increase in the distance between capillaries and muscle fibers [157,158]. Moreover, the MVU in the muscle of DMD patients showed marked structural alterations, and evidence suggested that DMD muscle is prone to impaired blood flow [9,153,159]. In a murine model of DMD, mice lacking dystrophin (mdx) exhibited decreased vascular density, and angiogenesis was markedly impaired in an mdx hindlimb-muscle ischemic mouse model [153,160,161]. Moreover, VEGF levels are significantly lower in mdx mice, and increasing VEGF expression by the administration of an adenovirus construct encoding VEGF or the transplantation of SCs expressing VEGF, improved vascularization as well as muscle regeneration [162,163]. Most interestingly, ECs and SCs isolated from mdx mice exhibit a reduced ability to promote angiogenesis and myogenesis, respectively, indicating that cross-talk exhibited in the EC–SC niche and critical to muscle capillarization and regeneration is impaired in DMD. Finally, as discussed by Latroche et al. [9], dystrophin deficiency also leads to a loss of muscle neuronal oxide synthase (nNOS) and thus the decreased production of freely diffusible NO. A defect in the vasoconstrictor response induced by muscle-derived NO in DMD patients, particularly during exercise, results in functional muscle ischemia. Accordingly, vascular dysfunction has been suggested as an underlying pathogenic mechanism of muscle injury in DMD and a target for the treatment of DMD patients ([9,153] for review).

In summary, a functional EC–SC niche is compromised in DMD, leading to improper capillarization and ischemia which compromise muscle regeneration and function. Although therapies to restore dystrophin remain important, targeting the vasculature to prevent ischemia and the increased demise of functional muscle is being actively explored.

## 7. Summary

Following the initiation of embryonic cell-signaling events that orchestrate the vascular and myogenic cell-lineage of MPCs from their common precursor, the dermatomyotome, ECs and myogenic precursors exhibit cross-talk and the expression of factors, e.g., VEGF, essential for the development of muscle, its vasculature and the pool of myogenic stem cells/SCs. EC–SC cross-talk maintains SC quiescence and localizes ECs to SCs to form an important EC–SC niche. Skeletal muscle development and function after birth reflects the interactive actions of ECs and muscle growth factors, primarily VEGF, as well as insulin. These interactions underpin myocyte capillarization, the activation of intracellular signaling mechanisms to elicit vasodilation important to blood flow dynamics and blood pressure, and the recruitment of capillaries to enhance surface area, required for the optimal transport of glucose and insulin across the endothelium into the interstitium and thereby available for uptake by, and action on, the myocyte. The dysfunctional interaction of ECs, muscle growth factors and insulin resulting from IR, and the loss of appropriate signaling, precipitate alterations in the MVU myocyte function that underpin vascular dysfunction/high blood pressure and T2DM. Studies in nonhuman primates indicate that the exposure of the fetus to elevated levels of estrogen in utero during the second half of pregnancy promotes fetal microvascularization and thus the normal development and function of the systemic vascular network and myofiber growth, required for eliciting insulin sensitivity and glucose tolerance and normal blood pressure after birth. Finally, EC–SC interactions underpin myofiber regeneration and the re-establishment of the vasculature and proper alignment of the MVU post-injury, a cross-talk and function which appears to be compromised in Duchenne muscular dystrophy.

## Figures and Tables

**Table 1:** Summary of the factors and cross-talk controlling skeletal-muscle myogenesis and vascular development in the embryo/fetus.

Description	Reference
Myogenic and vascular endothelial cells derived from myogenic progenitor cells (MPC) in the embryonic dermomyotome	Latroche et al., 2015 [9]; Kassar-Duchossoy et al., 2005 [13]; Mayeuf-Louchart et al., 2014 [17]; Ben-Yair et al., 2008 [20]
MPCs express paired box transcription factors (pax) 3 and/or 7, the forkhead box protein C2 transcription factor (foxc2) and thenotch trans-membrane receptor and downstream signaling	Kassar-Duchossoy et al., 2005 [13]; Lagha et al., 2008 [14]; Lagha et al., 2009 [15]; Buckingham and Rigby, 2014 [16]
Notch signaling controls pax 3/7: foxc2 gene equilibrium and thus vascular (low pax 3/7) myogenic (high pax3/7) lineage of MPCs	Mayeuf-Louchart et al., 2014 [17]
Myogenic cells undergo coordinated expression of myogenic regulatory factors (Mrf), 5, 4, D and G that underpin muscle formation	Latroche et al., 2015 [9]; Kassar-Duchossoy et al., 2005 [13]; Lagha et al., 2008 [14]; Ben-Yair and Kalcheim, 2008 [20]; Bentzinger et al., 2012 [29]
Endothelial cells (EC) express vascular endothelial growth factor (VEGF) that controls vascular development and vascularization/capillarization of developing muscle fibers	Ferrara et al., 1996 [22]; Ferrara, 1999 [23]
Myogenic-stem-cell pool comprised of pax 3/7 Mrf5/NOTCH satellite cells (SC) established	Lagha et al., 2008 [14]; Lagha et al., 2009 [15]; Ramirez de et al., 2022 [18]
VEGF induced activation of Delta-like 4 (DII4), a ligand for notch, establishes SC quiescence	Verma et al., 2018 [32]
VEGF signaling brings ECs close to SCs, forming an EC–SC niche important for muscle regeneration in adulthood	Christov et al., 2007 [7]; Latroche et al., 2015 [9]
Placental estradiol (E_2_) secreted into the fetus important for muscle capillarization during second half of pregnancy	Albrecht and Pepe, 1990 [39]; Albrecht et al., 2022 [40]

## Data Availability

Not applicable for this review.
